# Supplementary material for: Cardiac myosin-binding protein C interaction with actin is inhibited by compounds identified in a high-throughput fluorescence lifetime screen
Source: J Biol Chem. 2021 May 28;297(1):100840. doi: 10.1016/j.jbc.2021.100840 (PMC8233204; doi:10.1016/j.jbc.2021.100840)
Supplement: Tables S1–S4 and Figures S1–S3 [file mmc1.pdf]

Supplemental Material

Supplemental Tables

Table S1. Top 25 hits from Screen 1

	Rank	Row	Column	Actin-MyBP-C Drug Δτ (ns)	Actin Drug Δτ (ns)	Difference (ns)
**Suramin**	**1**	AD	29	0.088	-0.03	0.12
**NF 023**	**2**	R	21	0.058	-0.03	0.09
	**3**	R	42	0.018	-0.04	0.06
	**4**	T	27	0.008	-0.04	0.05
	**5**	R	7	0.013	-0.04	0.05
	**6**	Q	6	0.008	-0.04	0.04
**ATA**	**7**	K	9	0.008	-0.04	0.04
	**8**	X	2	-0.003	-0.04	0.04
	**9**	G	18	-0.006	-0.04	0.04
	**10**	D	34	0.011	-0.02	0.03
	**11**	Z	2	-0.010	-0.04	0.03
	**12**	R	22	-0.007	-0.04	0.03
	**13**	V	25	-0.008	-0.04	0.03
	**14**	D	40	0.017	-0.01	0.03
	**15**	R	30	-0.006	-0.04	0.03
	**16**	V	21	-0.006	-0.03	0.03
	**17**	S	37	-0.005	-0.03	0.03
	**18**	T	15	-0.010	-0.04	0.03
	**19**	X	7	-0.005	-0.03	0.03
	**20**	K	1	0.001	-0.03	0.03
	**21**	G	40	-0.005	-0.03	0.03
	**22**	X	39	-0.003	-0.03	0.03
	**23**	J	21	-0.014	-0.04	0.03
	**24**	R	46	-0.019	-0.05	0.03
	**25**	K	19	-0.017	-0.04	0.02

Top 25 hits from Screen 1 ranked include 3 Hits: Suramin (grey), NF 023 (blue), and ATA (red). Compounds are ranked for largest Difference (ns) due to effects on the change in lifetime (Δτ, ns) for actin +C0-C2 after subtracting the effect of compounds on Δτ for actin alone. Location of compound in row and column of wells is listed.

Table S2. Top 25 hits from Screen 2

	Rank	Row	Column	Actin-MyBP-C Drug $\Delta\tau$ (ns)	Actin Drug $\Delta\tau$ (ns)	Difference (ns)
**Suramin**	1	AD	29	0.15	0.00	0.16
	2	S	5	0.11	0.00	0.11
	3	T	30	0.10	0.00	0.10
	4	V	38	0.09	0.00	0.10
	5	S	41	0.09	0.00	0.09
	6	R	28	0.08	0.00	0.08
	7	T	4	0.04	-0.02	0.07
	8	R	12	0.05	-0.01	0.07
**NF 023**	9	R	21	0.07	0.01	0.06
	10	T	20	0.04	-0.02	0.06
**ATA**	11	K	9	0.06	0.00	0.06
	12	R	6	0.06	0.01	0.06
	13	R	4	0.03	-0.03	0.06
	14	T	37	0.05	0.00	0.06
	15	P	40	0.06	0.01	0.05
	16	T	17	0.05	0.00	0.05
	17	S	16	0.04	-0.01	0.05
	18	T	38	0.04	-0.01	0.05
	19	T	18	0.04	-0.01	0.05
	20	T	45	0.04	-0.01	0.05
	21	T	13	0.05	0.00	0.05
	22	R	32	0.03	-0.02	0.05
	23	T	19	0.03	-0.01	0.04
	24	W	44	0.04	-0.01	0.04
	25	S	20	0.02	-0.02	0.04

Top 25 hits from Screen 2 ranked include 3 Hits: Suramin (grey), NF 023 (blue), and ATA (red). Compounds are ranked for largest Difference (ns) due to effects on the change in lifetime ($\Delta\tau$, ns) for actin +C0-C2 after subtracting the effect of compounds on $\Delta\tau$ for actin alone. Location of compound in row and column of wells is listed.

Table S3. Top 25 hits from Sub-optimal Screen 3

	Rank	Row	Column	Actin-MyBP-C Drug Δτ (ns)	Actin Drug Δτ (ns)	Difference (ns)
**Suramin**	**1**	AD	29	0.18	-0.03	0.21
	**2**	M	35	0.17	-0.03	0.20
**ATA**	**3**	K	9	0.07	-0.03	0.10
	**4**	J	18	0.08	-0.02	0.10
	**5**	M	43	0.06	-0.03	0.09
	**6**	N	40	0.07	-0.03	0.09
	**7**	N	46	0.04	-0.05	0.09
	**8**	J	12	0.06	-0.02	0.09
**NF 023**	**9**	R	21	0.06	-0.02	0.08
	**10**	J	10	0.05	-0.03	0.08
	**11**	AA	47	0.03	-0.04	0.08
	**12**	AF	13	0.03	-0.03	0.07
	**13**	M	36	0.05	-0.01	0.07
	**14**	AA	18	0.02	-0.05	0.07
	**15**	K	45	0.05	-0.02	0.06
	**16**	J	13	0.03	-0.03	0.06
	**17**	L	10	0.05	-0.01	0.06
	**18**	N	30	0.04	-0.03	0.06
	**19**	N	34	0.02	-0.04	0.06
	**20**	AB	47	0.02	-0.04	0.06
	**21**	AB	12	0.04	-0.02	0.06
	**22**	AB	29	0.02	-0.04	0.06
	**23**	N	20	0.03	-0.03	0.06
	**24**	AF	19	0.02	-0.04	0.06
	**25**	J	38	0.03	-0.03	0.06

Top 25 hits from Sub-optimal Screen 3 ranked include 3 Hits: Suramin (grey), NF 023 (blue), and ATA (red). Compounds are ranked for largest Difference (ns) due to effects on the change in lifetime (Δτ, ns) for actin +C0-C2 after subtracting the effect of compounds on Δτ for actin alone. Location of compound in row and column of wells is listed.

Table S4. Top 25 hits from Sub-optimal Screen 4

	Rank	Row	Column	Actin-MyBP-C Drug Δτ (ns)	Actin Drug Δτ (ns)	Difference (ns)
**Suramin**	**1**	AD	29	0.13	0.04	0.09
**ATA**	**2**	K	9	0.07	0.00	0.07
	**3**	A	3	0.07	0.01	0.06
	**4**	A	1	0.06	0.01	0.05
	**5**	B	12	0.01	-0.03	0.04
	**6**	B	11	0.01	-0.03	0.04
	**7**	I	11	0.02	-0.02	0.04
	**8**	I	17	0.04	0.00	0.04
	**9**	J	21	0.03	0.00	0.04
	**10**	M	21	0.05	0.01	0.04
	**11**	L	15	0.05	0.01	0.04
	**12**	P	22	0.07	0.03	0.04
	**13**	A	4	0.03	0.00	0.04
	**14**	B	26	0.02	-0.01	0.03
	**15**	P	43	0.02	-0.01	0.03
	**16**	L	46	0.01	-0.02	0.03
	**17**	O	42	0.01	-0.02	0.03
	**18**	L	39	0.04	0.01	0.03
	**19**	O	5	0.06	0.03	0.03
	**20**	B	14	0.02	-0.01	0.03
**NF 023**	**21**	R	21	0.04	0.01	0.03
	**22**	P	28	0.03	0.00	0.03
	**23**	L	33	0.04	0.01	0.03
	**24**	M	19	0.04	0.01	0.03
	**25**	N	30	0.05	0.02	0.03

Top 25 hits from Sub-Optimal Screen 4 ranked include 3 Hits: Suramin (grey), NF 023 (blue), and ATA (red). Compounds are ranked for largest Difference (ns) due to effects on the change in lifetime (Δτ, ns) for actin +C0-C2 after subtracting the effect of compounds on Δτ for actin alone. Location of compound in row and column of wells is listed.

Supplemental Figures

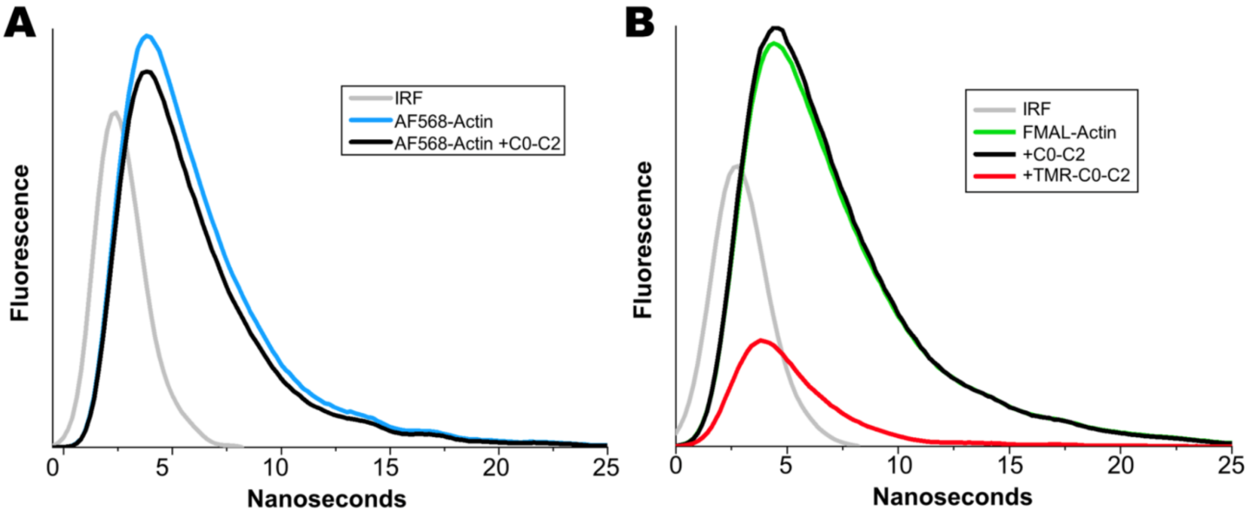

Figure S1. TR-F and TR-FRET waveforms as detected by direct waveform recording (DWR). ***(A)*** Waveforms of AF568-Actin ± C0-C2 (without normalization of fluorescence) detected in TR-F plate reader experiments. Normalized TR-F waveforms are found in **Fig. 2B** of main text. ***(B)*** Waveforms of donor-only, FMAL-Actin ± unlabeled C0-C2, and donor-acceptor, FMAL-Actin +TMR-C0-C2 (without normalization of fluorescence) detected in TR-FRET plate reader experiments. FRET occurs in donor-acceptor waveform (red) where both intensity and lifetime are reduced due to acceptor quenching of donor. Normalized TR-FRET waveforms are found in **Fig. 5B**. The instrument response function (IRF) is shown in grey.

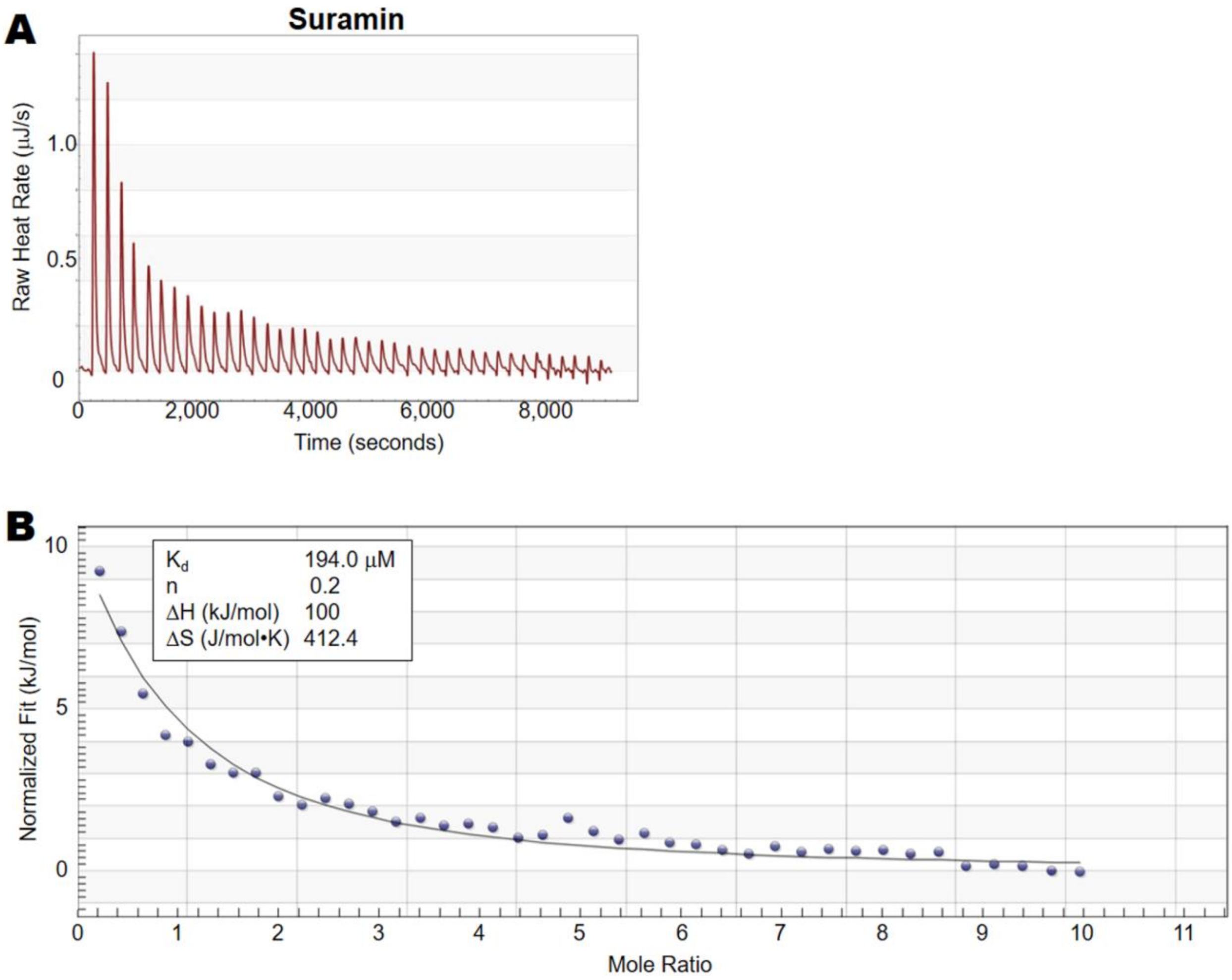

Figure S2. ITC of Suramin binding to actin alone. ***(A)*** Titration of 100 μM F-actin (in 350 μl) with 40 2.5 μl injections of 3,000 μM Suramin. ***(B)*** Fitted ITC binding curve for Suramin binding. Binding to actin was not apparent for NF023 or ATA (-0.2 < Raw Heat Rate (μJ/s) < 0.2), but weak interactions were observed for Suramin with actin alone (K_d ~200 μM).

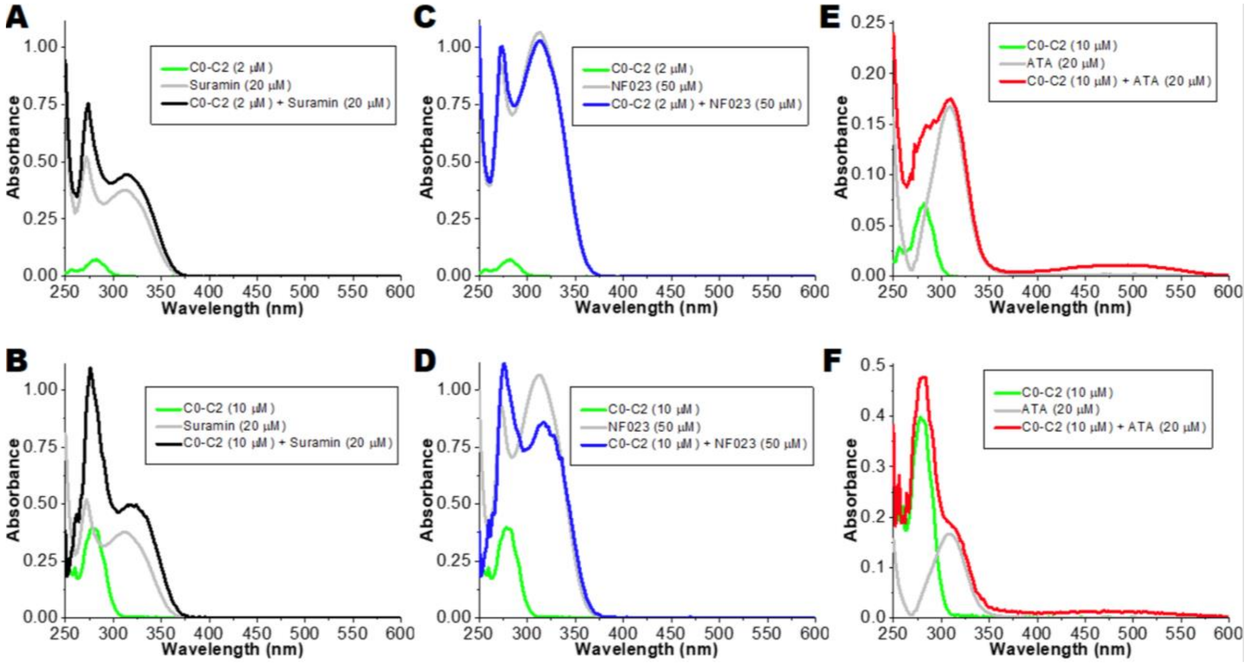

Figure S3. Superimposed wavelength scans of C0-C2, compound, and C0-C2 and compound mixtures after 20 minutes incubation: ***(A)*** 2 μM C0-C2, 20 μM Suramin, and 2 μM C0-C2 + 20 μM Suramin, ***(B)*** 10 μM C0-C2, 20 μM Suramin, and 10 μM C0-C2 + 20 μM Suramin, ***(C)*** 2 μM C0-C2, 50 μM NF023, and 2 μM C0-C2 + 50 μM NF023, ***(D)*** 10 μM C0-C2, 50 μM NF023, and 10 μM C0-C2 + 50 μM NF023, ***(E)*** 2 μM C0-C2, 20 μM ATA, and 2 μM C0-C2 + 20 μM ATA (4x zoom compared to A-D for Absorbance on y-axis), and ***(F)*** 10 μM C0-C2, 20 μM ATA, and 10 μM C0-C2 + 20 μM ATA (2x zoom compared to A-D for Absorbance on y-axis).
